# Spontaneous Transition of Spherical Coacervate to Vesicle‐Like Compartment

**DOI:** 10.1002/advs.202305978

**Published:** 2023-12-08

**Authors:** Hyunsuk Choi, Yuri Hong, Saeed Najafi, Sun Young Kim, Joan‐Emma Shea, Dong Soo Hwang, Yoo Seong Choi

**Affiliations:** ^1^ Department of Chemical Engineering and Applied Chemistry Chungnam National University Daejeon 34134 South Korea; ^2^ Division of Environmental Science and Engineering Pohang University of Science and Technology (POSTECH) Pohang 37673 South Korea; ^3^ Department of Chemistry and Biochemistry University of California Santa Barbara CA 93106 USA

**Keywords:** coacervation, intrinsically disordered proteins/regions, membrane‐less biomolecular compartments, vesicles‐like structure, vesicular condensates

## Abstract

Numerous biological systems contain vesicle‐like biomolecular compartments without membranes, which contribute to diverse functions including gene regulation, stress response, signaling, and skin barrier formation. Coacervation, as a form of liquid–liquid phase separation (LLPS), is recognized as a representative precursor to the formation and assembly of membrane‐less vesicle‐like structures, although their formation mechanism remains unclear. In this study, a coacervation‐driven membrane‐less vesicle‐like structure is constructed using two proteins, GG1234 (an anionic intrinsically disordered protein) and bhBMP‐2 (a bioengineered human bone morphogenetic protein 2). GG1234 formed both simple coacervates by itself and complex coacervates with the relatively cationic bhBMP‐2 under acidic conditions. Upon addition of dissolved bhBMP‐2 to the simple coacervates of GG1234, a phase transition from spherical simple coacervates to vesicular condensates occurred via the interactions between GG1234 and bhBMP‐2 on the surface of the highly viscoelastic GG1234 simple coacervates. Furthermore, the shell structure in the outer region of the GG1234/bhBMP‐2 vesicular condensates exhibited gel‐like properties, leading to the formation of multiphasic vesicle‐like compartments. A potential mechanism is proposed for the formation of the membrane‐less GG1234/bhBMP‐2 vesicle‐like compartments. This study provides a dynamic process underlying the formation of biomolecular multiphasic condensates, thereby enhancing the understanding of these biomolecular structures.

## Graphical abstract

This study shows the formation of a coacervation‐driven membrane‐less vesicle‐like structure using GG1234 and bhBMP‐2. A phase transition from spherical simple coacervates to vesicular condensates occurred upon the addition of bhBMP‐2 to GG1234 simple coacervates under acidic conditions, eventually leading to multiphasic vesicle‐like compartments with a gel‐like outer shell. They propose a dynamic process for the formation of these biomolecular structures.

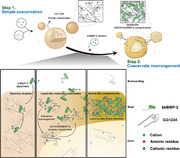

## 1 Introduction

As a fundamental unit of living organisms, cells organize their membrane‐less organelles (MLOs) based on biological condensation processes. Diverse structures of biomolecular condensates, such as liquid droplets, (liquid or solid) core‐ (liquid or solid) shells, and nonspherical and nonliquid condensates, are widely observed in biological systems.^[^ ^1^ ^]^ Adjustable compartmentalization of biomolecules in vitro could supply various membrane‐less architectures with different 3D structures and topologies, such as stress granules, P granules, Cajal bodies, paraspeckles, TIGER domains, and nucleoli. LLPS is essential for biomolecular condensation processes; however, the understanding of their formation dynamics and assembly processes is still in early stages despite their widespread occurrence in biological systems.^[^ ^2^ ^]^ Recently, the intriguing properties of MLOs have spurred the development of MLO‐inspired biomaterials based on intrinsically disordered proteins (IDPs) and regions (IDRs), offering great potential for applications such as biochemical reactors, synthetic biology, drug discovery, and drug delivery.^[^ ^3^ ^]^ Engineered synthetic biomolecular condensates provide biological insights into the fundamental mechanism of natural biomolecular condensates and offer a new strategy for biological design in biotechnological applications.^[^ ^4^ ^]^ Importantly, because LLPS mainly occurs in aqueous mixtures of different water‐soluble strongly interacting polymers, such as oppositely charged polyelectrolytes or in a solution of a single polymer with certain salts, LLPS from simple and complex coacervation has demonstrated great potential as a model of the formation of biomolecular condensates for the selective concentration of biomolecules.^[^ ^2^, ^5^ ^]^

Studying the formation of vesicular liquid substructures from coacervates is an important step in advancing our understanding of the selective colocalization and dissolution of condensate substructures. Several recent experiments have begun to shed light on these processes. Experiments involving the reentrant phase transition of RNA‐protein complexes demonstrated both the formation and dissociation of droplets and vesicular substructures as a result of complex coacervation and subsequent charge inversion, providing a feasible mechanism associated with droplet organelles in transcription.^[^ ^6^ ^]^ At high concentrations and disproportionate mixture compositions of nucleoprotein‐RNA complexes, stable hollow condensates with structural and functional similarities to lipid vesicles, such as molecular ordering, size‐dependent permeability, and selective encapsulation, were observed to form.^[^ ^7^ ^]^ Moreover, plant storage proteins such as soy glycinin, pea protein, and fava bean legumin formed hollow condensates via simple coacervation and their cavitation.^[^ ^8^ ^]^ The phase transition is driven by the interaction of electrostatic forces and hydrophobic and nonionic interactions, such as charge‐charge, cation‐π, dipole‐dipole, and π‐π stacking interactions, although the specific preferential interactions depend on biomolecular compositions and the exterior microenvironment.^[^ ^9^ ^]^ The multivalent nature of amphiphilic proteins and IDPs/IDRs has been regarded as one of the determining factors in the formation of vesicular condensates via LLPS.^[^ ^5^, ^10^ ^]^

**Figure 1:**
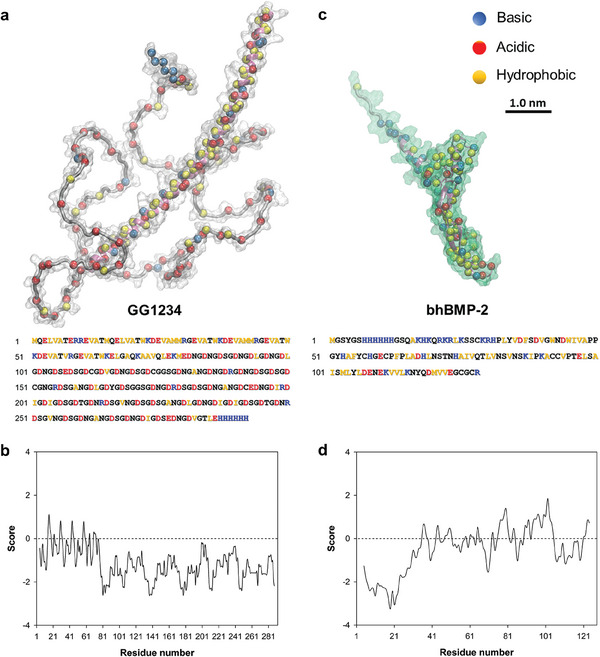
Primary structures of GG1234 and bhBMP‐2. a,c) The 3D structures of GG1234 and bhBMP‐2 proteins predicted by AlphaFold‐v2. The corresponding amino acid sequences of GG1234 and bhBMP‐2 are shown at the bottom. For both sequences, the yellow, blue, and red letters indicate the primary classification of the hydrophobic, basic, and acidic residues, respectively. b,d) Hydrophobicity scale profile of GG1234 and bhBMP‐2 obtained from the ProtScale tool (https://web.expasy.org/protscale/) using amino acid scale values from Kyte and Doolittle.

In this study, we constructed a vesicle‐like multiphasic condensate using an acidic protein named GG1234 (292 amino acids in length) and bioengineered human bone morphogenetic protein 2 (bhBMP‐2). GG1234 is a synthetic protein rich in glycine, aspartate, and serine that was inspired by acidic shell matrix proteins in biomineralization.^[^ ^11^ ^]^ Acidic proteins are commonly found in marine calcification and frequently identified in skeleton‐associated proteomes.^[^ ^12^ ^]^ Remarkably, GG1234 itself formed spherical simple coacervates, and the addition of bhBMP‐2 to the simple coacervates triggered stable vesicular structure formation due to interaction rearrangement through complex coacervation between GG1234 and bhBMP‐2. GG1234 has been shown to produce both simple and complex coacervates in various salt solutions under acidic conditions.^[^ ^11^ ^]^ Based on the secondary structure predicted by AlphaFold‐v2,^[^ ^13^ ^]^ GG1234 (a theoretical pI value of 3.58) is composed of a small structured region (1‐85; α‐helical and coil) and an aspartate/glycine‐rich IDR (86‐292) (**Figure** **1**a). Analysis of hydrophobicity using the Kyle and Doolittle scale and the ProtScale tool shows that the protein is amphiphilic (Figure 1b).^[^ ^14^ ^]^ In our investigation, bhBMP‐2 served as a trigger biomolecule for the formation of vesicle‐like structures. Human bone morphogenetic protein 2 (hBMP‐2), a structured protein with a theoretical pI value of 8.17, is a Food and Drug Administration (FDA)‐approved osteoinductive growth factor that has been clinically used as a bone graft substitute in the biomedical field due to its excellent bone regeneration properties.^[^ ^15^ ^]^ bhBMP‐2 was constructed by adding a hydrophilic IDR sequence to the N‐terminus of hBMP‐2 (Figure 1c,d). The N‐terminal IDR sequence of bhBMP‐2 contributed to the stable formation of GG1234/bhBMP‐2 vesicular condensates. Here, we describe a biomolecular vesicle‐like structure formation with a liquid interior and gel‐like surface based on complex coacervation within the shell region and internal phase separation of GG1234/bhBMP‐2 coacervate biomolecules. The results demonstrate that a protein‐based vesicle‐like structure can be spontaneously constructed via simple coacervation and subsequent complex coacervation. This investigation improves our understanding of the formation dynamics and assembly processes of membrane‐less vesicle‐like biomolecular compartments.

## 2 Results

### 2.1 The Transition of Spherical Coacervates to Vesicular Condensates in the GG1234/bhBMP‐2 System

**Figure 2:**
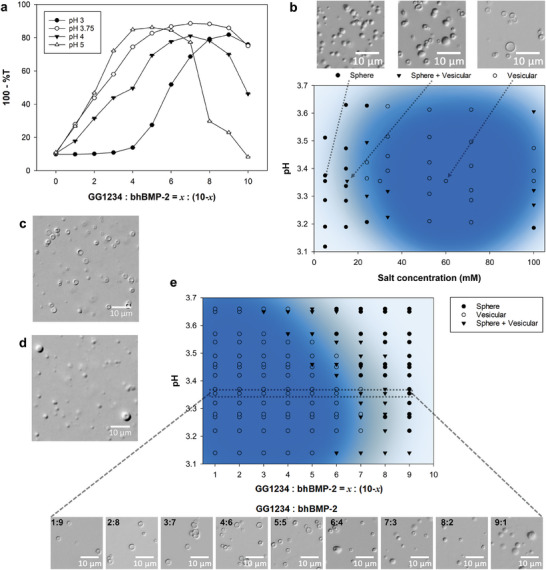
GG1234/bhBMP‐2 vesicular condensate formation in sodium acetate solution. a) Turbidimetric titration curve obtained by mixing different ratios of GG1234 and bhBMP‐2 in 50 mm sodium acetate solutions at different pH values. b) Morphological state diagram of the GG1234/bhBMP‐2 mixture in sodium acetate solutions at different pH values and salt concentrations for a 1:1 mixing ratio of GG1234 and bhBMP‐2. c) Optical microscopic image of the mixture of 2 mg mL^−1^ GG1234 simple coacervate solution (60 mM sodium acetate, pH 3.4) and 2 mg mL^−1^ bhBMP‐2 solution (60 mm sodium acetate, pH 3.4). d) Optical microscopic image of the mixture of 2 mg mL^−1^ GG1234 in distilled water and 2 mg mL^−1^ bhBMP‐2 solution (120 mm sodium acetate, pH 3.4). e) Morphological state diagram of different mixing ratios of GG1234 and bhBMP‐2 in 60 mm sodium acetate solutions at different pH values: spherical droplets (filled black circles with gray background), vesicular condensates (open circles with dark blue background), and the mixture of the spherical droplets and the vesicular condensates (filled inverted triangles with light blue background).

We observed a transition from spherically shaped GG1234 simple coacervates to vesicular condensates upon addition of the relatively positively charged bhBMP‐2 protein. To understand this transition phenomenon, we systematically investigated the formation of conventional spherical complex coacervates and vesicular condensates within the phase region in which GG1234 formed simple coacervates. **Figure** **2**a shows the turbidimetric titration results obtained by mixing different ratios of GG1234 and bhBMP‐2 within the pH range of 3–5 in a 50 mm sodium acetate solution. GG1234 simple coacervation occurred through charge neutralization combined with electrostatic, hydrophobic, and van der Waals forces.^[^ ^16^ ^]^ GG1234 formed simple coacervates at various sodium acetate concentrations under acidic conditions (pH 3–4).^[^ ^11^ ^]^ As expected, GG1234 simple coacervates were induced based on the pI values and the solubility of the protein, resulting in a turbidity increase when only GG1234 was present, as shown in Figure 2a (x = 10), whereas bhBMP‐2 alone did not induce any condensates in this condition (Figure 2a (x = 0)). Subsequent mixing of bhBMP‐2 with GG1234 changed the turbidity, and the mixing ratio for maximum turbidity increased with pH. Since the experimentally measured pKa values of the acidic residues (the average pKa values of Asp and Glu = 3.43 and 4.14) were in the above pH range,^[^ ^17^ ^]^ the degree of deprotonation of carboxyl residues in GG1234 depending on the solution pH likely controls the shape and charge densities of GG1234 in the solution, thereby modulating the overall charge density of the GG1234 protein and its coacervate phase. A small amount of precipitates was detected in the complex coacervation at pH 5 (data not shown), suggesting that the increase in the negative charge density of GG1234 destabilized the liquid phase. Because vesicular condensates were observed mainly in the pH range between 3 and 3.75 via phase‐contrast optical microscopy, a morphological state diagram of the complex coacervates was constructed from solution turbidity and optical microscopic images in this pH range as a function of salt concentration at room temperature with a 1:1 mixing ratio of GG1234 and bhBMP‐2 in a sodium acetate solution (Figure 2b). Spherical droplets, vesicular condensates, and a mixture of both components were observed depending on the salt and pH conditions. The main region of vesicular condensates (pH 3.2–3.5 and 40–80 mm sodium acetate) was effectively correlated to the optimal conditions for the formation of GG1234 simple coacervates (the dark blue area in Figure 2b). Remarkably, the simple mixing of bhBMP‐2 and GG1234 without the preceding GG1234 simple coacervation did not induce vesicular condensates. As an example, GG1234/bhBMP‐2 vesicular condensates were formed through complex coacervation by adding 2 mg mL^−1^ bhBMP‐2 to 2 mg mL^−1^ GG1234 simple coacervates at 60 mM sodium acetate (pH 3.4) (Figure 2c). However, the mixing of 2 mg mL^−1^ bhBMP‐2 (in 120 mm sodium acetate (pH 3.4)) and 2 mg mL^−1^ GG1234 (in distilled water; no simple coacervation) did not induce vesicular condensates; conventional complex coacervates of spherical morphology were mostly observed (Figure 2d). Subsequently, in dropwise mixing experiments using 2 mg mL^−1^ bhBMP‐2 (in 120 mm sodium acetate (pH 3.4)) and 2 mg mL^−1^ GG1234 (in distilled water; no simple coacervation), only conventional spherical complex coacervates were observed (Figure S1, Supporting Information). The preceding formation of GG1234 simple coacervates was essential for the formation of GG1234/bhBMP‐2 vesicular condensates. Furthermore, the addition of hBMP‐2 instead of bhBMP‐2 to GG1234 simple coacervates at the same condition exhibited inefficient formation of these vesicular condensates, leading to the predominant formation of conventional spherical complex coacervates (Figure S2, Supporting Information). Although a minor core‐shell structure was partly observed upon hBMP‐2 addition, the morphological changes were largely less pronounced than the GG1234/bhBMP‐2 vesicular condensate formation. When the relatively basic bhBMP‐2 was added to the simple coacervates, complex coacervation of the two proteins occurred, and a shape transition was observed. Similarly, a phase diagram was also obtained in calcium acetate solution for the same mixture of GG1234 and bhBMP‐2 (Figure S3, Supporting Information), but the region of vesicular condensates was shifted to a slightly increased pH. Divalent Ca^2+^ rather than monovalent Na^+^ could contribute to the shift in the optimal charge neutralization condition.^[^ ^16^, ^18^ ^]^ As expected, its area was consistent with the optimal region of GG1234 simple coacervate formation in the calcium acetate solution. In addition, when a phase diagram of spherical droplets and vesicular condensates from the complex coacervation was prepared with the different mixing ratios of GG1234 and bhBMP‐2 in this region, the vesicular condensates were optimally detected in a low proportion of GG1234 between a pH range of 3.2 and 3.5 (Figure 2e). The number of vesicular condensates increased with GG1234, but conventional spherical droplets gradually formed in a high proportion of the GG1234 wt.% region (dark circles and inverted triangles in Figure 2e). The vesicular condensates could not develop without GG1234 simple coacervates, and a certain concentration of bhBMP‐2 per GG1234 simple coacervate appeared to be required.

**Figure 3:**
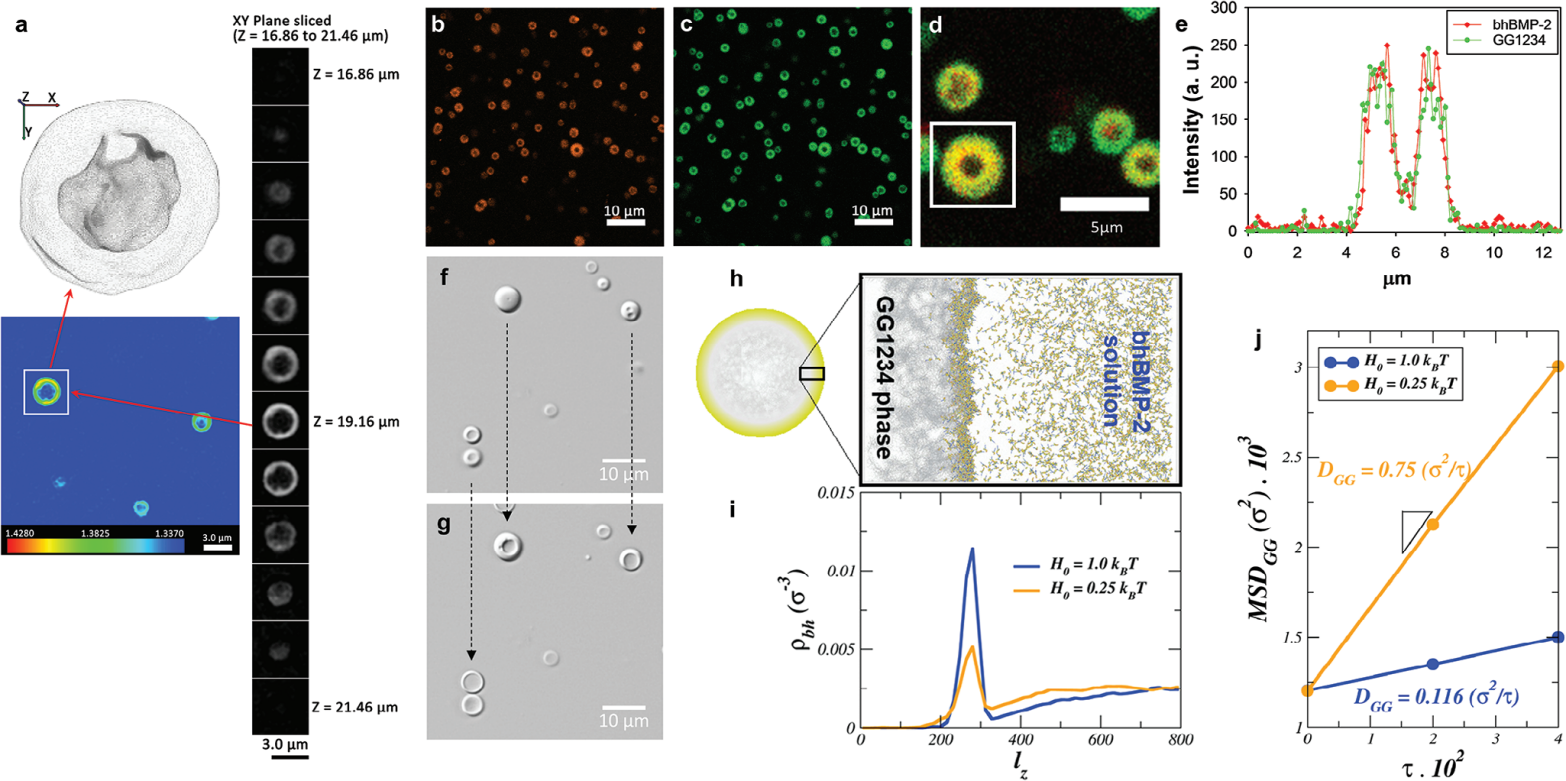
GG1234/bhBMP‐2 vesicular condensate structure. a) 3D label‐free RI image of the vesicular condensates using optical diffraction tomography. The right image shows the cross‐sectional slices of a single vesicular condensate RI distribution along the x‐y plane. The left images show the overall RI distribution of the cross‐sectional slice at Z = 19.16 µm (left bottom) and a 3D rendered image of the RI distribution (left top). Confocal fluorescence microscopy images of the vesicular condensates obtained by rhodamine‐labeled b) bhBMP‐2 and c) FITC‐labeled GG1234. d) Dual‐color fluorescence image of the labeled vesicular condensates. e) Fluorescence intensity profile of the cross‐sectional line of the labeled vesicular condensate (white box in (d)). f) The formation of GG1234/bhBMP‐2 vesicular condensates 20 s after the addition of bhBMP‐2 to GG1234 simple coacervates. g) The formation of GG1234/bhBMP‐2 vesicular condensates 5 min after the addition of bhBMP‐2 to GG1234 simple coacervates. h) Molecular dynamics snapshot of the GG1234 dense slab in coexistence with the bhBMP‐2 solutions. i) Density profile of bhBMP‐2 across the simulation box in (h), which indicates the accumulation of bhBMP‐2 at the interface of the GG1234 slab. j) The MSD of the GG1234 protein center of mass within the dense slab of GG1234 at high (H_0_ = 1.0 k_B_T) and low (H_0_ = 0.25 k_B_T) hydrophobicity scales; the corresponding diffusion coefficients are obtained by calculating the slope of the MSD.

**Figure** **3**a shows a 3D label‐free refractive index (RI) image of the GG1234/bhBMP‐2 vesicular condensates at 60 mM sodium acetate (pH 3.4) using optical diffraction tomography,^[^ ^19^ ^]^ demonstrating its core‐shell structure. The RI values of a protein solution were linearly proportional to the protein concentration.^[^ ^20^ ^]^ The RI values of the core were similar to those of the exterior dilute‐phase solution in the cross‐sectional slice image of the 3D RI (Figure 3a), indicating that the protein concentrations in the core and the exterior solution could be similar. Upon the protein concentration analysis in the surrounding dilute phase, the concentration decreased to ≈25%. As expected, when we labeled bhBMP‐2 with rhodamine (Figure 3b) and GG1234 with fluorescein 5(6)‐isothiocyanate (FITC) (Figure 3c), two‐color confocal fluorescence microscopy showed that both proteins were distributed in the shell of the condensates, but they were significantly diluted in the core region (Figure 3d). Because both proteins were distributed evenly in the shell region (Figure 3e), the preexisting GG1234 simple coacervates could be disrupted by the interaction between GG1234 and bhBMP‐2, and the GG1234 protein contributed to the formation of the shell structure with bhBMP‐2. In this manner, the initial spherical morphology of GG1234 simple coacervates gradually changed to vesicular condensates after introducing bhBMP‐2 into the simple coacervates (Figure 3f,g; Movie S1, Supporting Information). The initial core‐shell structure was relatively rapidly formed within 20 s after adding bhBMP‐2 to GG1234 simple coacervates. Subsequently, the diameter of the core region gradually increased while the shell thickness steadily decreased over a few minutes. Additionally, the size of the vesicular condensates appeared to depend on the size of the simple coacervates. After the formation of simple coacervates, the average size of GG1234 simple coacervates in 60 mm sodium acetate (pH 3.4) was found to be 2.33 ± 0.42 µm after 1 min and 3.26 ± 1.28 µm after 10 min (Figure S4, Supporting Information). The size of GG1234 simple coacervates increased and appeared to gradually precipitate over time, which could be a common feature of liquid coacervates.^[^ ^21^ ^]^ When bhBMP‐2 was applied to the simple coacervates obtained after 1 and 10 min, the average sizes of the formed GG1234/bhBMP‐2 vesicular condensates also increased to 2.55 ± 0.71 µm and 4.88 ± 1.26 µm, respectively (Figure S4, Supporting Information). The size of GG1234/bhBMP‐2 vesicular condensates exhibited a relatively rapid initial growth followed by a gradual increase over time (Figure S5, Supporting Information). It seems that the size of GG1234/bhBMP‐2 vesicular condensates can be adjusted by the addition of dissolved bhBMP‐2 at various times after the formation of GG1234 simple coacervates. In addition, we used the Alphafold‐v2 database with the recently released learning code^[^ ^13^ ^]^ to predict the structures of GG1234 and bhBMP‐2 (Figure 1a,c). The prediction suggested that the proteins were amphiphilic, consisting of a flexible IDR and a structured region containing a significant hydrophobic region. The hydrodynamic sizes are ≈4.5 nm for GG1234 and 2.1 nm for bhBMP‐2. Coarse grained (CG) molecular dynamics (MD) simulation in implicit solvent initiated from the structures of GG1234 and bhBMP‐2 predicted via Alphafold‐v2 showed that the increase in the effective hydrophobicity enhanced bhBMP‐2 assembly on the surface of GG1234 simple coacervates (Figure 3h,i; Movie S2, Supporting Information) (see the Experimental Section for the simulation details). In general, due to electrostatic screening at high salt concentrations or low pH conditions, the hydrophobic interaction between GG1234 and bhBMP‐2 protein increases. We use the strength of the attractive hydrophobic interaction between the hydrophobic residues of the GG1234 and bhBMP‐2, as a proxy to implicitly tune the salt concentration or solution pH condition. The MD simulations revealed that the bhBMP‐2 proteins gradually accumulate on the GG1234 slab (which primarily represents the GG1234 simple coacervate) due to hydrophobic interactions. The higher hydrophobicity strength that can be achieved at higher salt concentrations or lower pH conditions (where the electrostatic interactions are mainly screened) can lead to increased and more rapid complexation of the two proteins on the interface. Moreover, the MD simulation indicated that the helical segment of GG1234 was responsible for the formation of pseudo‐scaffold constructs of 4–5 nm diameter size voids within the GG1234 simple coacervates at low pH or high hydrophobicity conditions (Movie S3, Supporting Information). The neutralized acidic residues of GG1234 at low pH lead the hydrophobicity of the residues dominating, and the voids enable the diffusion of bhBMP‐2 protein into the GG1234 simple coacervates, leading to the formation of complex coacervates in the shell region. Conversely, at high pH, the GG1234 protein within the condensate displays heightened mobility (Movie S4, Supporting Information), attributed to the absence of a structured condensate observed in the low pH system. The combination of GG1234's increased mobility and the absence of voids at high pH poses a hindrance to BMP‐2 penetration. Collectively, these results indicated that a vesicular structure was mainly formed through the complex coacervation of GG1234 and bhBMP‐2 on the surface of GG1234 simple coacervates; this resulted in the colocalization and assembly of both proteins at the interface between the condensate core and dilute phase, due to the increased hydrophobicity and attractive interaction of GG1234 and bhBMP‐2 at low pH conditions.

### 2.2 GG1234/bhBMP‐2 Vesicular Condensates as a Model for Membrane‐Less Vesicle‐Like Biomolecular Compartments

**Figure 4:**
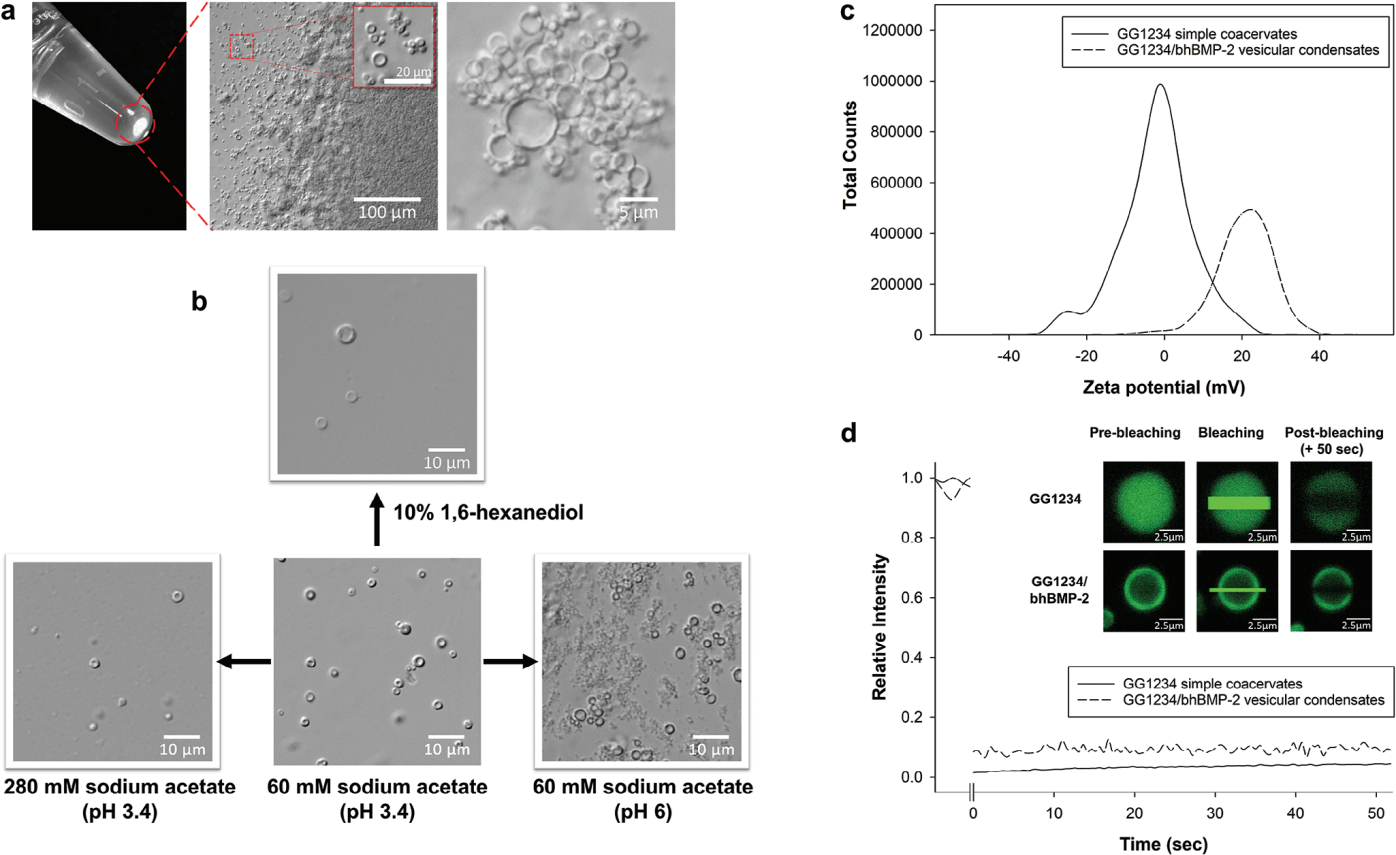
Accumulation and conservation of individual GG1234/bhBMP‐2 vesicular condensates. a) Gradual accumulation of the individual GG1234/bhBMP‐2 vesicular condensates on the bottom. b) Exposure of the constructed GG1234/bhBMP‐2 vesicular condensates in 60 mm sodium acetate (pH 3.4) into a higher salt solution (280 mm sodium acetate, pH 3.4), a higher pH solution (60 mm sodium acetate, pH 6.0), and 10% 1,6‐hexanediol. c) Zeta potential profile of the GG1234 simple coacervates and GG1234/bhBMP‐2 vesicular condensates. d) Fluorescence recovery after photobleaching the GG1234 simple coacervates and GG1234/bhBMP‐2 vesicular condensates.

The GG1234/bhBMP‐2 vesicular condensates gradually accumulated on the bottom without merging the condensates (**Figure** **4**a). However, conventional liquid coacervate droplets thermodynamically favor fusion into larger condensates to reduce the free energy of the system.^[^ ^22^ ^]^ In this manner, when lysozyme was used as a polyelectrolytic basic partner (pI = 11.35 and 129 amino acids)^[^ ^23^ ^]^ instead of bhBMP‐2, GG1234/lysozyme complex coacervate droplets fused to a dense liquid phase at the bottom over time (Figure S6, Supporting Information). Additionally, the GG1234/bhBMP‐2 vesicular structure was conserved when the constructed condensates were exposed to other solution conditions that disfavored vesicular structure formation. For example, the vesicular structure obtained from 60 mm sodium acetate solution (pH 3.4) was conserved in both exposure to 280 mm sodium acetate (pH 3.4) and 60 mm sodium acetate (pH 6.0) (Figure 4b), although some spherical droplets and irregular precipitates were also observed with increasing salt concentration and pH. Moreover, the structure was still observed upon the addition of 1,6‐hexanediol, but the number was decreased predominantly due to the dissolution of the hydrophobic liquid condensates (Figure 4b). Some liquid‐like vesicular condensates could be transferred to a solid‐like form and not dissolved in the 1,6‐hexanediol solution as 1,6‐hexanediol has been shown to disrupt the hydrophobic interactions of liquid condensates but not solid condensates.^[^ ^9b^ ^]^ In passive microrheology, the viscosity of GG1234 simple coacervates was determined to be 56.41 Pa·s in 60 mm sodium acetate solution (pH 3.4) (Figure S7, Supporting Information). However, the viscosity measurement of GG1234/bhBMP‐2 vesicular condensates was not successful because probe nanoparticles could not penetrate the structure, where the edge might be in a solid phase that does not allow merging. Moreover, the average zeta potential of GG1234 simple coacervates in 60 mm sodium acetate (pH 3.4) was −2.03 ± 8.49 mV (Figure 4c), indicating that charge neutralization occurred. However, the average zeta potential of GG1234/bhBMP‐2 vesicular condensates in 60 mM sodium acetate (pH 3.4) was 20.6 ± 7.37 mV (Figure 4c). The positive surface charges on the surface of the vesicular condensate as well as edge solidification likely prevent them from merging. Previously, stable hollow condensate topology was also obtained at disproportionate mixture compositions of disordered RNA‐binding proteins and RNA complexes with a substantial excess net charge (negative in excess RNA or positive in excess protamine).^[^ ^7^ ^]^ In addition, because bhBMP‐2 (128 amino acids) is a structured protein with an IDR sequence and is relatively neutral (pI = 8.17), the GG1234/bhBMP‐2 condensate phase could be driven by the relatively high contribution of hydrophobic and nonionic interactions.

Additionally, when the molecular mobility of GG1234 in GG1234 simple coacervates and GG1234/bhBMP‐2 vesicular condensates was monitored by fluorescence recovery after photobleaching (FRAP), the fluorescence of Alexa488‐labeled GG1234 in both condensates was not recovered after photobleaching (Figure 4d). Noncovalent interactions among GG1234 proteins and highly viscoelastic properties could not allow the diffusion of GG1234 within GG1234 simple coacervates. We obtained the GG1234 protein diffusion coefficient within the GG1234 slab by calculating the slope of the GG1234 center of mass mean square displacement (MSD) time evolution. The MD simulation also corroborated that the GG1234 protein within the condensate is highly immobile at low pH (Figure 3j; Movies S3 and S4, Supporting Information). Upon adding bhBMP‐2 to the simple coacervates of GG1234, the interaction of the neutralized and highly viscoelastic GG1234 with the more hydrophobic bhBMP‐2 forms the GG1234/bhBMP‐2 vesicular structure. Moreover, increased hydrophobicity induces the gelation of the formed GG1234/bhBMP‐2 liquid‐like condensates,^[^ ^24^ ^]^ resulting in the accumulation of individual vesicle‐like condensates (Figure 4a). At pH 3.4, GG1234 consists of 15.5% charged residues and 84.5% nonpolar and polar neutral amino acids, and bhBMP‐2 consists of 20.9% charged residues and 79.1% nonpolar and polar neutral amino acids. Notably, the proteins involved in MLO formation are typically composed of ≈10–20% charged residues and 80–90% predominantly polar neutral and aromatic residues,^[^ ^5^ ^]^ whereas most complex coacervates as viscoelastic liquids are composed of highly charged peptides or polymers with a high local charge concentration.^[^ ^5^ ^]^ From these results, we deduced that GG1234/bhBMP‐2 vesicular condensates had a vesicle‐like structure with a liquid core and a gel‐like rigid shell, which could provide insight into the formation of biomolecular vesicle‐like MLOs.

## 3 Discussion

**Figure 5:**
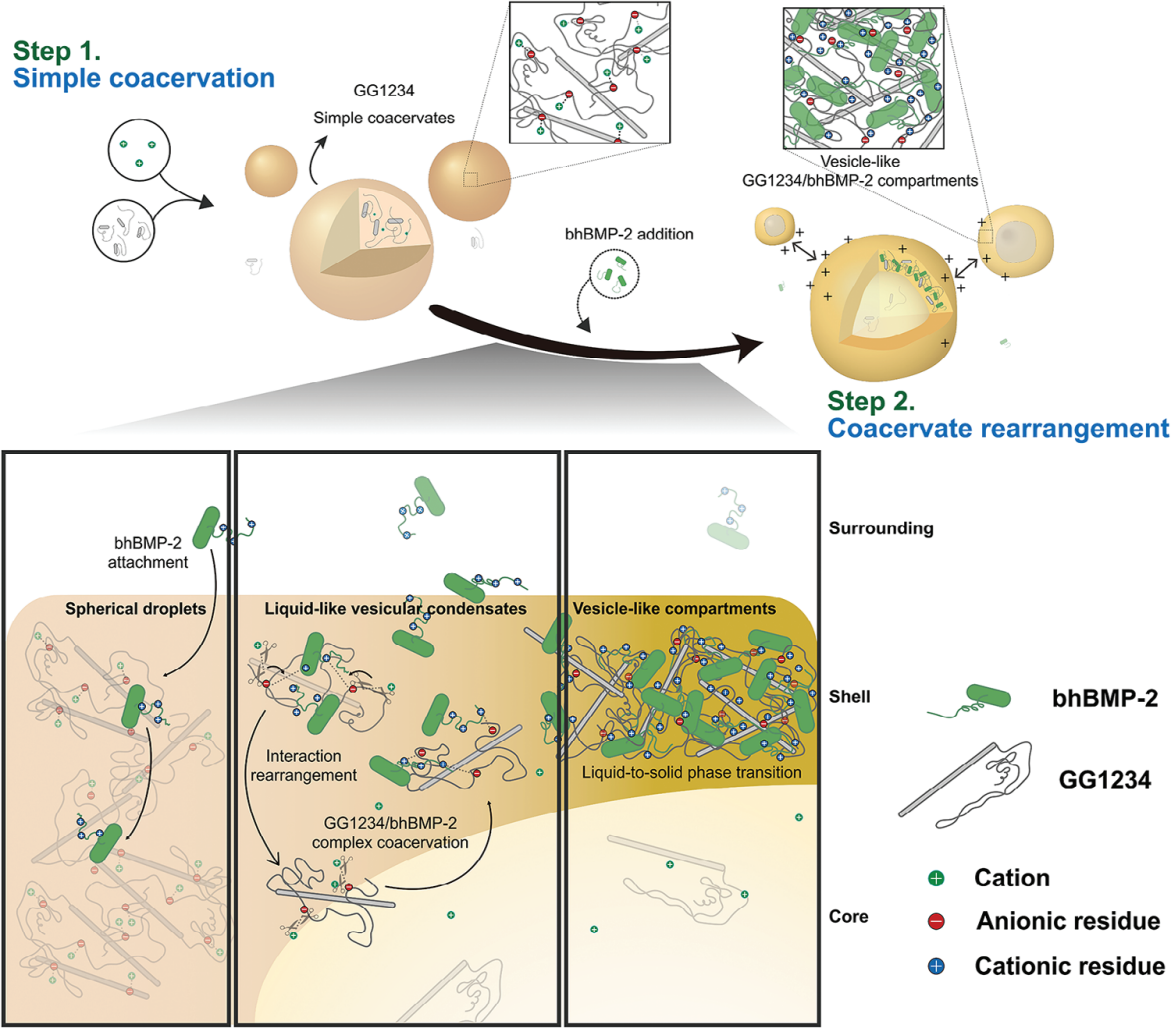
Schematic illustration of a proposed mechanism of the formation of membrane‐less vesicle‐like GG1234/bhBMP‐2 compartments. Vesicle‐like structure formation is processed through the following steps: 1) GG1234 spherical droplets are formed by simple coacervation in the absence of bhBMP‐2. 2) The addition of bhBMP‐2 triggers coacervate rearrangement via noncovalent interaction rearrangement from GG1234 simple coacervation to GG1234/bhBMP‐2 complex coacervation. Then, the liquid‐like GG1234/bhBMP‐2 complex coacervate is transformed to a gel‐like GG1234/bhBMP‐2 complex with a vesicle‐like structure due to the increased hydrophobicity.

A large number of MLOs have been recently found both in prokaryotes and in the nucleus and cytoplasm of eukaryotes.^[^ ^2a^ ^]^ MLOs can be liquids, solids, or gels that organize the cells, and are composed of condensates of protein, nucleic acid, or both. These biomolecular condensates contribute to numerous functions, including gene regulation, stress response, signaling, and skin barrier formation.^[^ ^1^, ^25^ ^]^ However, the formation of vesicular multiphasic compartments in a representative MLO microstructure remains poorly understood. Our study indicated that the self‐rearrangement of coacervate biomolecules could construct membrane‐less vesicle‐like biomolecular compartments with a liquid core and a gel‐like shell (**Figure** **5**). In our model system, the two proteins GG1234 and bhBMP‐2 were composed of a relatively hydrophobic structured region and a hydrophilic IDR region, respectively. Amphiphilic proteins that have the ability to form vesicles with a membrane structure could be components of MLOs.^[^ ^26^ ^]^ The simple coacervation of GG1234 in the absence of bhBMP‐2 was first performed as an initial step for the subsequent formation of vesicle‐like GG1234/bhBMP‐2 vesicular condensates. Spherical GG1234 simple coacervate droplets formed due to the decreased solvation of hydrophobic residues and intramolecular self‐association of charged residues (7.6% negatively charged, 7.9% positively charged) in the acidic salt condition.^[^ ^5^ ^]^ Then, the addition of bhBMP‐2 to the GG1234 coacervate solution triggered the formation of GG1234/bhBMP‐2 vesicular condensates. Polymer‐rich liquid‐phase droplets (GG1234 simple coacervates) functioned as a precursor, and subsequent internal phase separation of the GG1234 liquid‐phase via the addition of bhBMP‐2 from the bulk solution evolved the spherical coacervates into vesicle‐like compartments (GG1234/bhBMP‐2 complexes) at the coacervate phase interface.^[^ ^27^ ^]^ The hydrophobic and relatively cationic bhBMP‐2 proteins attached to the highly viscoelastic GG1234 simple coacervate surface as a result of the hydrophobic and attractive electrostatic interaction between GG1234 and bhBMP‐2. The intermolecular interaction destabilized the simple coacervates and stimulated GG1234/bhBMP‐2 complex coacervation at the simple coacervate surface consistent with GG1234 and bhBMP‐2 forming spherical complex coacervates in the absence of GG1234 simple coacervates (Figure 2). Additionally, hBMP‐2, in place of bhBMP‐2, failed to induce the core–shell structure effectively (Figure S2, Supporting Information). Protonated histidine residues of the N‐terminal IDR sequence of bhBMP‐2 could enhance the interaction between GG1234 and bhBMP‐2 in the shell region, impeding the penetration of bhBMP‐2 into the core region and thereby contributing to the stable formation of the GG1234/bhBMP shell structure. Then, the positive surface charge of the GG1234/bhBMP‐2 complex (Figure 4c) could function as a membrane‐like barrier and induce a water‐filled structure within the complex coacervates. Similarly, water‐filled vacuoles were developed through osmotic pressure differences derived from membrane formation on the droplet surface.^[^ ^28^ ^]^ Eventually, the addition of bhBMP‐2 into the GG1234 simple coacervates spontaneously reorganized the multiphasic condensate structure and transformed the spherical droplets into vesicular condensates. In addition, the localized GG1234/bhBMP‐2 complex coacervates within the rim transferred into a gel‐like structure due to the decreased hydration. Coacervates, as dense viscoelastic liquids, can transfer to gels and solids due to the decreased hydration from the enhanced hydrophobicity.^[^ ^26^, ^29^ ^]^ A liquid‐to‐solid phase transition in GG1234/bhBMP‐2 vesicular condensates potentially occurred due to the increased hydrophobicity within the rim. Moreover, biomolecular liquid condensates do not uniformly convert to a solid phase during condensate aging, and the liquid‐to‐solid transition is initiated from the interface and propagates toward the center of the condensates.^[^ ^30^ ^]^ This irreversible phase transition stabilized some vesicle‐like structures without dissolution to the liquid phase, even though the pH and salt concentration were changed into spherical condensate formation or single liquid phase regions (Figure 4b). In this manner, when GG1234/bhBMP‐2 spherical droplets precipitated over time, the droplets did not merge as a dense liquid but rather separately accumulated on the bottom similar to the vesicular condensates, and individual spherical granules were observed after drying the sample (Figure S8, Supporting Information).

Here, we show a vesicle‐like compartment formation model based on phase transitions from coacervates: the formation of spherical droplets, the subsequent rearrangement of spherical droplets to vesicular condensates, and the liquid‐to‐solid phase transition of the rim of the vesicular condensates. Coacervate condensates can be viable candidates for MLOs; they are spontaneously formed through the abiotic physical phenomenon of LLPS and potentially display or mimic characteristics commonly present in living cells.^[^ ^26^ ^]^ However, conventional spherical coacervates do not have a membrane, which is prone to coalescence and has limited stability.^[^ ^26^ ^]^ Liquid‐to‐solid phase transition to form a vesicle‐like structure is a feature of MLOs and can be correlated to the fundamental mechanism of cellular organization and processes,^[^ ^5^, ^31^ ^]^ deviating from the nature of coacervates.^[^ ^5^ ^]^ In this aspect, this study indicates that the interaction between highly viscoelastic simple coacervates and hydrophobic biomolecules, such as GG1234 simple coacervates and bhBMP‐2, is involved in LLPS and liquid‐to‐solid phase transition for the formation of membrane‐less vesicle‐like biomolecular compartments. We propose a potential mechanism for the formation of multiphasic compartments through the spontaneous transition of homogenous coacervates to vesicular condensates, thereby advancing our understanding of these biomolecular structures. Furthermore, in biotechnological applications, BMP‐2 products often require the reconstitution of lyophilized BMP‐2 using acidic solutions before use, owing to the low solubility and significant aggregation of hBMP‐2 in physiological conditions.^[^ ^32^, ^33^ ^]^ Lowering the carrier pH to acidic conditions can be a significant factor in the BMP‐2 formulation, reducing the effective protein dose and enhancing bone formation in clinical applications.^[^ ^34^ ^]^ We anticipate that the formulation of GG1234/bhBMP‐2 vesicle‐like compartments holds potential applications for delivering BMP‐2 and other biomolecules to enhance bone regeneration. The exploration of these phase transitions leading to vesicular assemblies of biomolecular complexes is expected to greatly contribute to our understanding of subcellular organization and facilitate biotechnological applications of vesicular biomolecular substructures.

## 4 Experimental Section

### Preparation of GG1234 and bhBMP‐2

The synthetic protein GG1234 was produced in *Escherichia coli* based on a previously described method.^[^ ^11^ ^]^ Briefly, *E. coli* BL21 (DE3) cells containing the GG1234 expression vector were cultured at 37 °C in 50 mL of Luria‐Bertani (LB) medium with ampicillin (50 µg mL^−1^) until the optical density (OD) reached ≈0.8. Isopropyl β‐D‐1‐thiogalactopyranoside (IPTG) (0.05 mm) was applied to induce protein overexpression, and then the cells were incubated at 20 °C for 20 h. The cells were disrupted in 5 mL lysis buffer (100 mm NaH_2_PO_4_, 10 mm Tris‐base, 10 mM imidazole, 8 m urea; pH 8.0) using an ultrasonic cell disrupter (Sonosmasher ULH‐700S; Ulsso High‐Tech Co., Korea) at 30% power with a 3‐seconds pulse on and 17‐seconds cooling period between each burst. The protein was obtained by Ni‐nitrilotriacetic acid (Ni‐NTA) affinity purification using wash buffer (50 mm NaH_2_PO_4_, 300 mm NaCl, 20 mm imidazole; pH 8.0) and elution buffer (50 mm NaH_2_PO_4_, 300 mm NaCl, 300 mm imidazole; pH 8.0). Finally, the protein was prepared in distilled water by dialysis using a 15 mL centrifugal filter (Amicon Ultra, 10 kDa cutoff; Merck Millipore, USA).

The plasmid vector for the expression of bhBMP‐2 was prepared from pET‐28b (Merck Millipore, USA) by introducing the additional genetic sequence of Gly‐Ser‐Tyr‐Gly‐Ser‐His‐His‐His‐His‐His‐His‐His‐Gly‐Ser into the N‐terminus of the hBMP‐2 sequence. Glycine/histidine‐rich oligopeptides may serve as IDR sequences for LLPS, and the use of a 6x‐histidine has proven effective in protein purification, ensuring homogeneity without batch‐to‐batch variation.^[^ ^35^, ^36^ ^]^ The expression plasmid was transferred into *E. coli* BL21 (DE3), and the cells were grown in 50 mL of LB medium with kanamycin (10 µg mL^−1^) at 37 °C until the OD reached 0.8. bhBMP‐2 purification was conducted similarly to the purification of GG1234 via Ni‐NTA affinity chromatography using lysis buffer (100 mm NaH_2_PO_4_, 10 mm Tris‐base, 10 mm imidazole, 8 m urea; pH 8.0), wash buffer (75 mm NaH_2_PO_4_, 5 mm Tris‐base, 150 mm NaCl, 15 mm imidazole, 4 m urea; pH 8.0), and elution buffer (50 mm NaH_2_PO_4_, 300 mm NaCl, 3 m imidazole; pH 5.0). Finally, the protein was prepared in 60 mm sodium acetate (pH 3.4) by changing the buffer using a 15 mL centrifugal filter.

### GG1234 Simple Coacervation and GG1234/bhBMP‐2 Complex Coacervation

GG1234 (4 g L^−1^) was initially prepared at room temperature in distilled water, and sodium acetate solutions and calcium acetate solutions of various concentrations (0–200 mm) at different pH values (pH 3.0–6.0) were added to the GG1234 solution at a 1:1 ratio for the simple coacervation of GG1234 (final concentration: 2 g L^−1^). In addition, bhBMP‐2 was dissolved in the same buffer solution used to form the simple coacervates. Vesicular condensates were fabricated by mixing GG1234 simple coacervates and bhBMP‐2. Moreover, bhBMP‐2 in various salt solutions was directly mixed with GG1234 in distilled water as control experiments. The turbidity of each sample was determined by the previously described method through absorbance measurement,^[^ ^11^ ^]^ and the morphology of the constructed condensates was observed using phase‐contrast optical microscopy (Eclipse Ni‐U; Nikon Instruments Inc., USA).

### Optical Diffraction Tomography

Optical diffraction tomography (ODT) was performed using a commercial ODT microscope (HT‐2H; Tomocube Inc., Korea) based on Mach–Zehnder interferometry equipped with a digital micromirror device (DMD). A coherent monochromatic laser (532 nm) was divided into two paths, a reference, and a sample beam, using a 2 × 2 single‐mode fiber coupler. A 3D RI tomogram was reconstructed from multiple 2D holographic images acquired from 49 illumination conditions, a normal incidence, and 48 azimuthally symmetric directions with a polar angle (64.5°). The DMD was used to control the angle of an illumination beam impinging onto the sample. The diffracted beams from the sample were collected using a high numerical aperture (NA) objective lens (NA = 1.2, UPLSAP 60XW, Olympus). The off‐axis hologram was recorded via a complementary metal–oxide–semiconductor image sensor (FL3‐U3‐13Y3MC, FLIR Systems Inc.). GG1234/bhBMP‐2 vesicular condensates were prepared using simple coacervates of GG1234 (2 g L^−1^) and bhBMP‐2 (2 g L^−1^) in 60 mm sodium acetate (pH 3.4), imaged and visualized using a commercial software (TomoStudio, Tomocube Inc.).

### Fluorescence Labeling of GG1234 and bhBMP‐2

FITC was purchased from Sigma–Aldrich and used to label GG1234 based on the instructions provided. bhBMP‐2 was labeled with a two‐fold molar excess of NHS‐rhodamine (Thermo Fisher Scientific, USA). Briefly, the labeling reaction was carried out at room temperature for 2 h. Unreacted dye was removed and buffer was exchanged using a centrifugal filter (Amicon Ultra, 3 kDa cutoff; Merck Millipore, USA).

### Confocal Fluorescence Microscopy

Coacervates were prepared using the protein solutions containing 1% fluorescence‐labeled protein. Images of the coacervates were obtained via confocal microscope (LSM800; Zeiss, Germany) equipped with a 63x/NA 1.4 Oil objectives. The acquired images were analyzed using ImageJ.

### Passive Microrheology for Viscosity Measurement

PEG‐passivated beads with a diameter of 0.5  µm (L3280, Sigma, Saint Louis, MO) were added to GG1234 simple coacervates prepared in 60 mm sodium acetate solution. The beads embedded in the GG1234 simple coacervates (*n* = 13) were imaged at specific intervals using a confocal microscope (FV3000, Olympus, Japan) at 20 °C. The MSD was calculated using MATLAB software (Mathworks, MA). The diffusion constant (*D*) was acquired by fitting an averaged log‐log plot of MSD versus lag time (*τ*) with MSD = 4*Dτ^α^* in the region of diffusive exponent (*α*) ≈ 1; specifically, the slope of the curve on a log (MSD)‐log (lag time) plot was used. The viscosity (*η*) was calculated from the Stokes−Einstein equation as follows: $D\;=\;\frac{k_{B}T}{6\pi \eta a}$, where *k_B_* is the Boltzmann constant, *T* is the temperature, and *a* is the radius of bead.

### Zeta Potential Measurement

GG1234 simple coacervates and GG1234/bhBMP‐2 vesicular condensates were prepared by the described methods. Zeta potential measurements were performed using a particle size analyzer with a Zetasizer Nano ZS (Malvern Instruments, USA).

### FRAP

FRAP was conducted using a confocal microscope (Leica TCS SPX, Leica, Germany) with the coacervate sample that contained 1% Alexa 488 labeled GG1234 protein. The labeled protein and its coacervate samples were prepared and carried out in the manner described in the Methods. Partial droplet bleaching was conducted to understand diffusion in coacervate building blocks. Bleaching regions were selected and bleached with a 488 nm argon laser at 100% power during the bleaching step. At the pre/post‐bleaching steps, less than 20% of the 488 nm argon laser power was used to excite Alexa‐488 fluorescence dye. The images were acquired with a size of 512 × 512 pixels every 0.522 s. The intensity data were collected with a focus on the bleached area. The fluorescence relative intensity profile plot was calculated using the highest intensity value of each data group as 1.0. The zero point on the *x*‐axis was converted based on when the postbleaching process started.

### 3D Structure Prediction and MD Simulation

MD simulations were performed starting from the structure of the GG1234 and bhBMP‐2 proteins predicted by AlphaFold‐v2.^[^ ^13^ ^]^ A coarse‐grained representation of the proteins was used, in which each residue was modeled by a positively, negatively, or charge neutral bead. Details of the simulations and the models can be found in the Supporting Information. Briefly, all the monomers exclude each other with a soft‐core Lenard Jones potential. Additionally, the charged monomers interact through a long range coulombic electrostatic interaction in an implicit solvent whose background dielectric constant ∈ was ≈10 to model the low pH condition. In particular, the hydrophobic residues that were determined according to the primary classification of nonpolar amino acids, attract each other through a double‐well potential, where the second depth represents the dehydration attraction (see Figure S9, Supporting Information). The study first construct a dense slab that consists of ≈3000 GG1234 proteins and embed the same number of bhBMP‐2 proteins in the dilute phase; the counter ions were included to satisfy the charge balance condition. All the simulations were performed at constant volume with Langevin thermostats of temperature *T* = 1.0, using the large‐scale atomic/molecular massively parallel simulator package.^[^ ^33^ ^]^ The equations of motion were integrated with the time step Δt = 0.005τ, where τ is the MD time unit, τ = σ (m/ε)^1/2^, m is the monomer mass, σ is the monomer diameter and ε is the unit of energy. Each simulation was performed for 5.10^6^ τ. To assure the thermodynamics convergence, the first 2.10^6^ τ was discarded when the analyses were performed.

## Conflict of Interest

The authors declare no conflict of interest.

## Author Contributions

H.C., D.S.H., and Y.S.C. conceived the experiments. H.C., Y.H., S.Y.K., D.S.H, and Y.S.C. performed the experiments and analyzed the data. S.N. and J.‐E.S. designed and performed simulations, and analyzed the data. H.C, S.N, J.‐E.S., D.S.H., and Y.S.C. wrote the manuscript.

## Supporting information

Supporting Information

Supplemental Movie S1

Supplemental Movie S2

Supplemental Movie S3

Supplemental Movie S4

## Data Availability

The data that support the findings of this study are available from the corresponding author upon reasonable request.
