# Supplementary material for: Spontaneous Transition of Spherical Coacervate to Vesicle‐Like Compartment
Source: Adv Sci (Weinh). 2023 Dec 8;11(7):2305978. doi: 10.1002/advs.202305978 (PMC10870063; doi:10.1002/advs.202305978)
Supplement: Supplementary file 1 — Supporting Information [file ADVS-11-2305978-s005.pdf]

ADVANCED SCIENCE
Open Access

Supporting Information

for *Adv. Sci.*, DOI 10.1002/advs.202305978

Spontaneous Transition of Spherical Coacervate to Vesicle-Like Compartment

Hyunsuk Choi, Yuri Hong, Saeed Najafi, Sun Young Kim, Joan-Emma Shea, Dong Soo Hwang* and Yoo Seong Choi**

Supporting Information for “Spontaneous transition of spherical coacervate to vesicle-like compartment”

Hyunsuk Choi, Yuri Hong, Saeed Najafi, Sun Young Kim, Joan-Emma Shea,* Dong Soo Hwang,* and Yoo Seong Choi*

*Corresponding author.

H. Choi, S. Y. Kim, Prof. Y. S. Choi

Department of Chemical Engineering and Applied Chemistry, Chungnam National University, Daejeon 34134, Korea

E-mail: chs930227@gmail.com (H. Choi), dydn5588@mabik.re.kr (S. Y. Kim), biochoi@cnu.ac.kr (Y. S. Choi)

Dr. Y. Hong, Prof. D. S. Hwang

Division of Environmental Science and Engineering, POSTECH, Pohang 37673, Korea

E-mail: yrhong@postech.ac.kr (Y. Hong), dshwang@postech.ac.kr (D. S. Hwang)

Dr. S. Najafi, Prof. J.-E. Shea

Department of Chemistry and Biochemistry, University of California, Santa Barbara, CA 93106, USA

E-mail: snajafi@ucsb.edu (S. Najafi), shea@chem.ucsb.edu (J.-E. Shea)

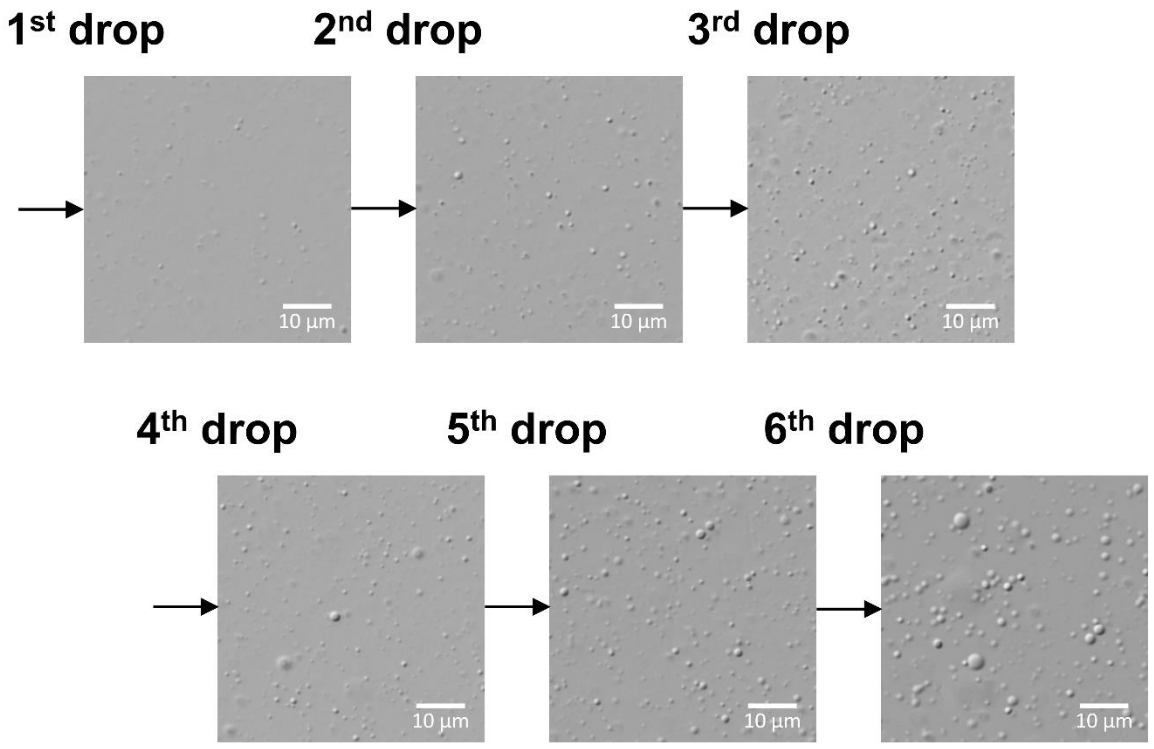

Figure S1. Dropwise mixing of GG1234 in distilled water to bhBMP-2 in 120 mM sodium acetate (pH 3.4). 3 µL (equivalent to one drop volume) of 2 mg/mL GG1234 in distilled water was gradually added dropwise into 18 µL of 2 mg/mL bhBMP-2 in 120 mM sodium acetate (pH 3.4). In order to ensure consistent final concentrations, distilled water was additionally added to each dropwise sample.

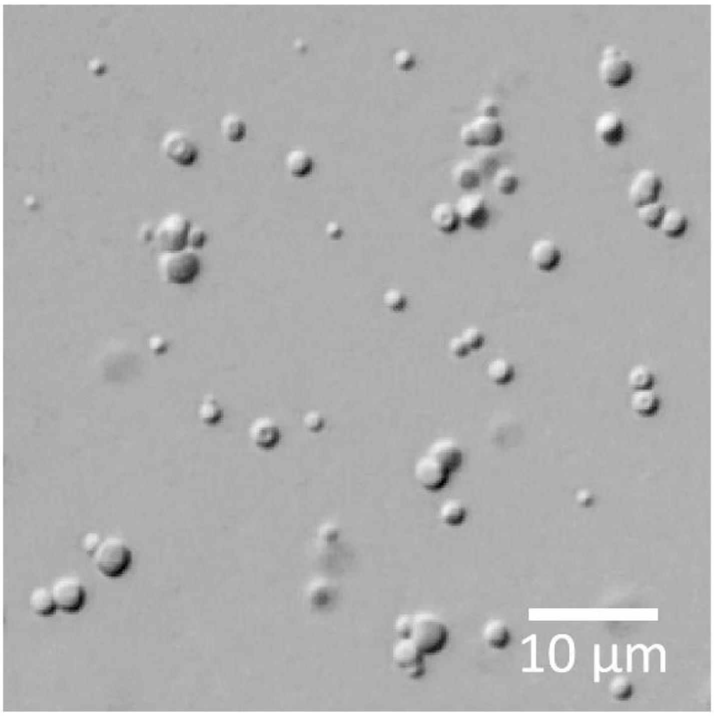

Figure S2. Optical microscopic image of the mixture of 2 mg/mL GG1234 simple coacervate solution (60 mM sodium acetate, pH 3.4) and 2 mg/mL hBMP-2 solution (60 mM sodium acetate, pH 3.4).

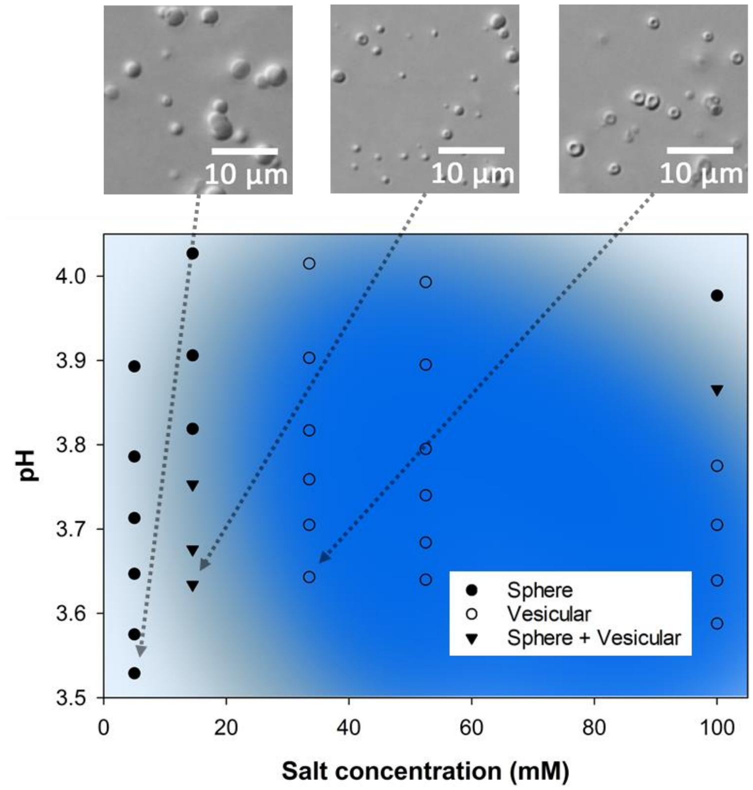

Figure S3. GG1234/bhBMP-2 vesicular condensate formation in calcium acetate solution. Morphological state diagram of GG1234/bhBMP-2 mixture was constructed in calcium acetate solutions with different pHs and salt concentrations for 1:1 mixing ratio of GG1234 and bhBMP-2: spherical droplets (filled black circles with gray background), vesicular condensates (open circles with bark blue background), and the mixture of the spherical droplets and the vesicular condensates (filled inverted triangles with light blue background).

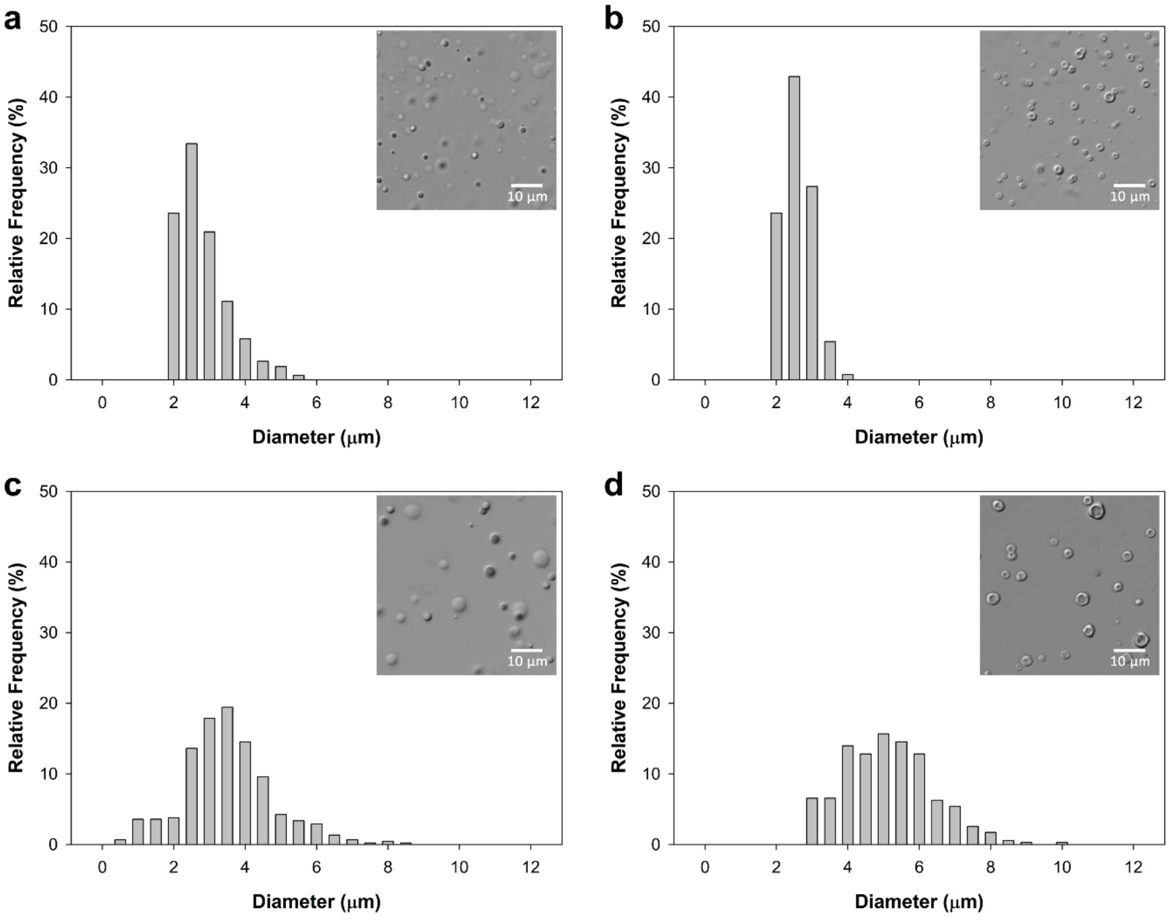

Figure S4. Size distribution of GG1234/bhBMP-2 vesicular condensates induced from the GG1234 simple coacervates with different sizes. Size distribution of GG1234 spherical droplets after 1 min from the GG1234 simple coacervation in 60 mM sodium acetate (pH 3.4) (**a**) and G1234/bhBMP-2 vesicular condensates subsequently induced from the GG1234 simple coacervates (**b**). Size distribution of GG1234 spherical droplets after 10 min from the GG1234 simple coacervation in 60 mM sodium acetate (pH 3.4) (**c**) and G1234/bhBMP-2 vesicular condensates subsequently induced from the GG1234 simple coacervates (**d**).

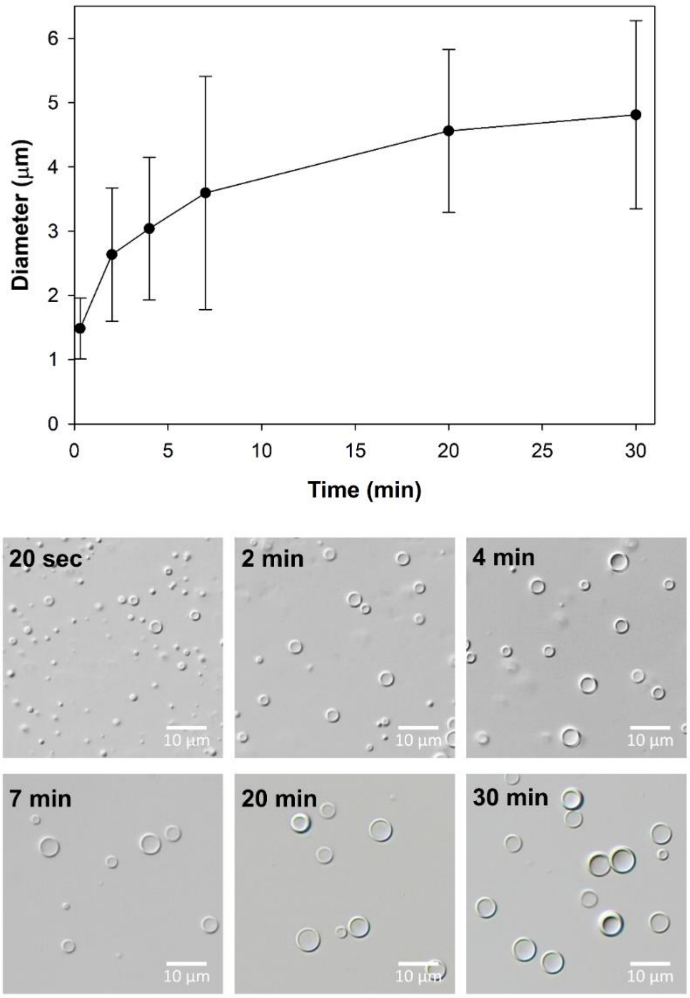

Figure S5. Size distribution of GG1234/bhBMP-2 vesicular condensates induced by the addition of dissolved bhBMP-2 to the GG1234 simple coacervates obtained at various times

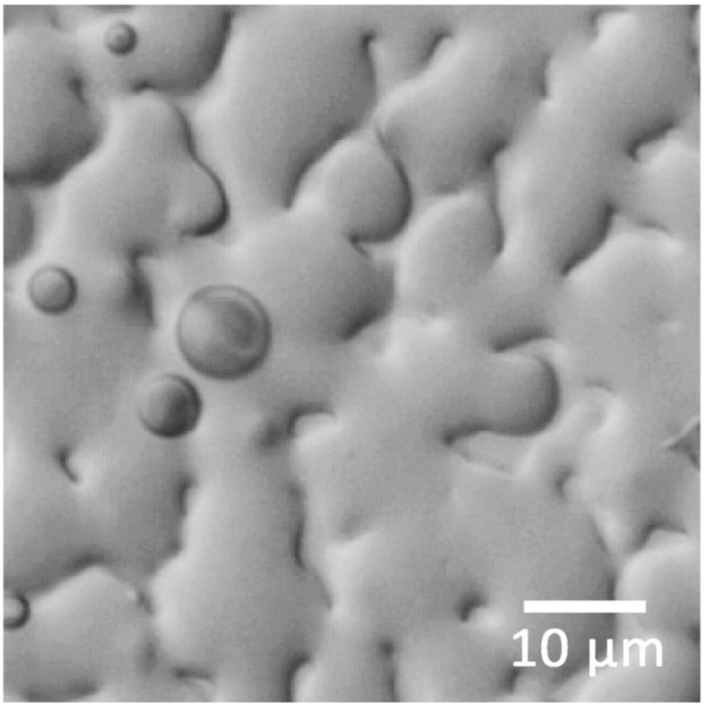

Figure S6. Fusion of the GG1234/lysozyme complex coacervate droplets to a dense liquid phase.

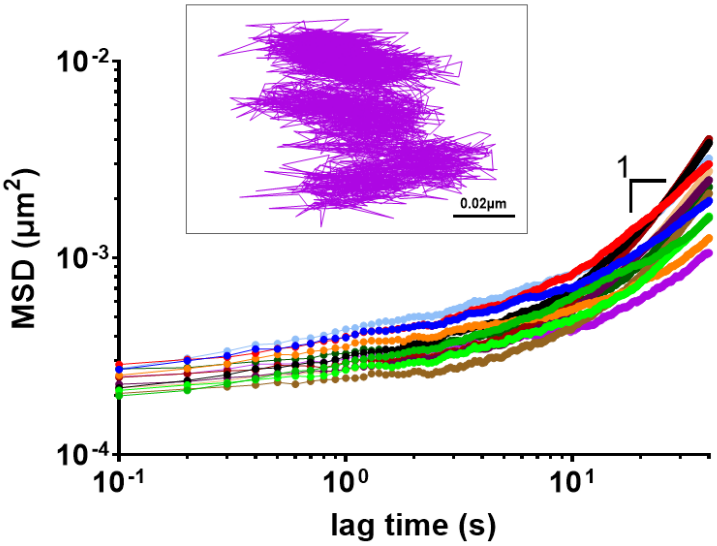

Figure S7. Mean squared displacement (MSD) data for individual beads (n=13) from GG1234 simple coacervates. A diffusion coefficient, D, is obtained by fitting the MSD data to $\langle MSD \rangle = 4D\tau^{\alpha}$, where $\alpha = 1$ for viscous liquid. The GG1234 simple coacervate viscosity, η, is determined through the Stokes-Einstein relation.

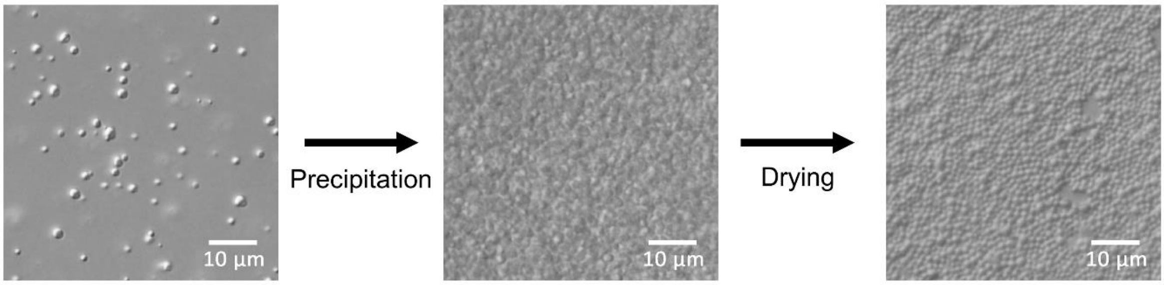

Figure S8. Accumulation and drying of GG1234/bhBMP-2 spherical droplets. GG1234/bhBMP-2 spherical droplets were obtained by the complex coacervation of GG1234 and bhBMP-2 in 5 mM sodium acetate solution (pH 3.4). The spherical condensates were accumulated on the glass surface for 24 hr, and the sample was dried at room temperature after removing the salt solution.

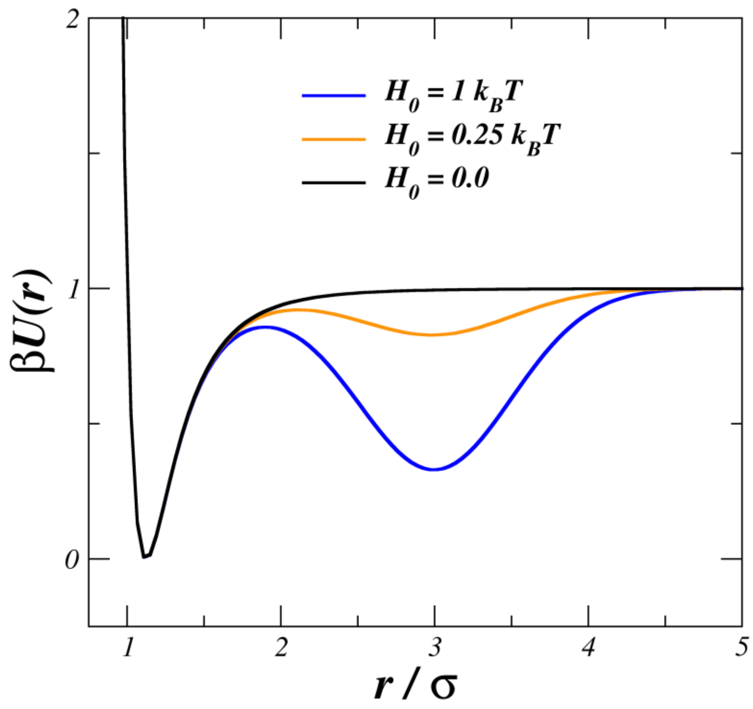

Figure S9. The coarse-grained hydrophobic potential of interactions between residues. The H_0 indicates the strength of the hydrophobic interactions. β is inverse k_BT and σ is the coarse-grained residues diameter.

The non-electrostatic potential of interaction between the coarse-grained hydrophobic residues is given by: $\beta U(r)_{hydrophob} = 4\epsilon_0 \left[\left(\frac{\sigma}{r}\right)^{12} - \left(\frac{\sigma}{r}\right)^{6} \right] + 1 - H(r)$

The attractive interaction is given by:

$$H(r) = \frac{H_0}{\delta(2\pi)^{0.5}} \exp\left(-\frac{(r - r_0)^2}{2\delta^2}\right)$$

Where $\delta = 0.6\,\sigma$ and $r_0 = 3\,\sigma$.

Movie S1. GG1234/bhBMP-2 vesicular condensate formation. This video shows the formation procedure of GG1234/bhBMP-2 vesicular condensates after the addition of bhBMP-2 to spherical GG1234 simple coacervates.

Movie S2. Coarse grained molecular dynamics simulation in implicit solvent from the structure of GG1234 and bhBMP-2 proteins predicted by AlphaFold-v2. This movie illustrates that BMP-2 protein promptly accumulates on the surface of the GG1234 coacervate and after that, the BMP-2 protein starts to penetrate into the coacervate through a rather slow and rate-limited process.

Movie S3. The formation of a pseudo-scaffold construct of 4-5 nm diameter size voids in the molecular dynamics simulation. At low pH, the GG1234 protein exhibits significant immobility due to the formation of a pseudo-scaffold structure with voids, which intriguingly promotes BMP-2 penetration.

Movie S4. The motion of GG1234 proteins with the vesicular condensate in the molecular dynamics simulation. At high pH, the GG1234 protein within the condensate displays heightened mobility, attributed to the absence of a structured condensate observed in the low pH system.
